# Longitudinal relationship between hip displacement and hip function in children and adolescents with cerebral palsy: A scoping review

**DOI:** 10.1111/dmcn.16175

**Published:** 2024-11-21

**Authors:** Ailish Malone, Giorgia Tanner, Helen P. French

**Affiliations:** ^1^ School of Physiotherapy, Royal College of Surgeons in Ireland University of Medicine and Health Sciences Dublin Ireland; ^2^ School of Medicine, Royal College of Surgeons in Ireland University of Medicine and Health Sciences Dublin Ireland

## Abstract

**Aim:**

To identify, describe, and synthesize available evidence on the longitudinal relationship between hip displacement and hip function, using the International Classification of Functioning, Disability and Health (ICF) framework, in children and adolescents with cerebral palsy (CP) aged up to 18 years.

**Method:**

Five databases were searched systematically from inception to May 2022. Study and sample characteristics, and hip displacement and hip function measures, mapped to the ICF domains, were extracted for narrative synthesis.

**Results:**

Twenty‐nine studies were included: four longitudinal registry‐based studies; 12 prospective studies; 12 retrospective studies; and one randomized controlled trial. Sample size ranged from 11 to 267. Twenty‐seven (93%) studies entailed an intervention: surgery (*n* = 16); rehabilitation (*n* = 2); nerve block or botulinum neurotoxin A injection (*n* = 4); and combined surgery and injection (*n* = 2). Twenty‐six studies (90%) reported outcomes at the body structure and function and impairment domain of the ICF; 17 (59%) reported outcomes in the activity domain; and three (10%) included participation measures. The most common hip displacement measure was Reimers' migration percentage (79%).

**Interpretation:**

Because of the inclusion of interventions in most studies, and the preponderance of retrospective studies, the relationship between hip displacement and hip function in CP is unclear. More high‐quality prospective evidence on the natural history of hip displacement, and its effect on function, is needed to improve population‐wide screening of children with CP.

## Graphical abstract

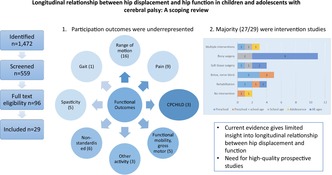

Plain language summary: https://onlinelibrary.wiley.com/doi/10.1111/dmcn.16208

**Abbreviations**
CPCHILD: Caregiver Priorities and Child Health Index of Life with Disabilities
ICF: International Classification of Functioning, Disability and Health
RMP: Reimers' migration percentage
ROM: range of motion

**What this paper adds**

- Current evidence provides limited insight into the longitudinal relationship between hip displacement and function in children and adolescents with cerebral palsy.
- Participation outcomes are underrepresented, with only three studies reporting outcomes with a participation component.
- High‐quality longitudinal, prospective, and registry studies are needed.
- Longitudinal data on hip function are needed to understand its natural history.

Cerebral palsy (CP) is ‘a group of permanent disorders of the development of movement and posture, causing activity limitations that are attributed to non‐progressive disturbances that occurred in the developing fetal or infant brain’.^1^ It is the most common motor disability of childhood, with a prevalence of 1.6 per 1000 live births.^2^ Approximately one in three children with CP develop hip displacement,^3^, ^4^, ^5^ with the risk being highest in children aged 2 years and 5 years who have severe gross motor function limitation and either spastic or dyskinetic CP.^5^, ^6^ Hip displacement refers to lateral displacement of the femoral head out of the acetabulum, which can vary from partial displacement (subluxation) to complete displacement of the femoral head from under the acetabulum (dislocation).

Hip development in childhood is the result of a delicate interplay between mechanical influences as a child progresses through typical childhood milestones. Development of the acetabular shape is dependent on its interaction with the spherical head of the femur. Formation of the ball‐and‐socket joint continues to take place in early childhood, up to 8 years, which is considered an important age for prognosis in paediatric hip disorders.^7^ Children with CP typically have normal hips at birth but progress to subluxation and dislocation later in life. Contributing factors include overactive hip flexor and adductor muscles, as well as poor use of hip extensors and hip external rotators,^8^ which lead to proximal femoral valgus deformity and anteversion. These muscle imbalances are especially prevalent in non‐ambulatory children who are unable to develop muscle to stabilize the hips, therefore increasing the risk of hip displacement.^5^, ^9^ This lateral displacement usually occurs gradually, where muscle tone and spasticity worsen with growth, placing a constant force on the hip as it develops, thus increasing the likelihood of further displacement as a child ages.^10^ Hip displacement poses a challenge to children and caregivers, not only because it is painful in itself and affects the ability to sit, stand, or walk,^5^ but because it can lead to pressure ulcers, difficulty in perineal hygiene, and reduced quality of life.^11^, ^12^

Radiographs are the most common imaging modality used to detect displacement. Reimers' migration percentage (RMP) is the most commonly used parameter and considered the criterion standard to assess displacement.^13^ It is defined as the width of the uncovered femoral head relative to the width of the femoral head.^14^ An RMP of less than 33% is generally deemed low‐risk for dislocation from longitudinal studies; anything over 40% is high‐risk and 100% indicates a fully dislocated hip.^9^, ^15^, ^16^ Other measures used to determine displacement include the acetabular index, or lateral centre‐edge angle, which measures acetabular dysplasia. Measures of femoral neck‐shaft or head‐shaft angle and anteversion may be also used to describe proximal femur morphology. Increased neck‐shaft and head‐shaft angles and anteversion are associated with increased hip displacement.^17^, ^18^

The impact of hip displacement on children with CP must be considered beyond biomechanical factors. The International Classification of Functioning, Disability and Health (ICF) is a framework used to systematically describe function at the level of the body, the individual, and wider society, also referred to as body structure, activities, and participation respectively.^19^ For a heterogeneous disability like CP, the ICF provides a deeper insight into how a child will function in the real world. A recent scoping review found that, in longitudinal studies of CP, activity was the most commonly reported ICF domain; 89% of studies reported the Gross Motor Function Classification System (GMFCS) level.^20^ The GMFCS classifies children into five categories of motor function from levels I to V, with each additional level requiring more mobility aids and support than the preceding level.^21^ A linear correlation between GMFCS level and hip dislocation incidence has been found.^22^, ^23^ Children functioning in GMFCS level V are 2.5 to 3 times more likely to develop a dislocated hip than those functioning in levels III and IV,^22^ emphasizing the importance of ambulation on the development of hip displacement. However, with high incidence of non‐motor impairments in CP, such as intellectual disability and hearing and visual impairments, the GMFCS does not give a complete view of how a child with CP will participate in the world around them. Therefore, for a more comprehensive overall view of function, the ICF framework is recommended, whereas the GMFCS can be seen as a broad stratification of risk for hip dislocation in CP.

Because of the complexity of CP and the changes occurring within the musculoskeletal system as a child grows and develops, the hip is potentially in a state of flux until adulthood. Previous studies showed a cross‐sectional association between hip displacement and pain and quality of life, whereby children with higher hip displacement had more pain^24^ and poorer quality of life.^25^, ^26^ To date, no comprehensive synthesis of the literature on the longitudinal relationship between hip displacement and hip function has been undertaken in children and adolescents with CP up to the age of 18 years. This creates a gap in knowledge about how hip function changes over time in children and adolescents with CP until they reach adulthood. It is important to identify the impact of hip displacement on hip function, and particularly to determine if there are critical periods in childhood and adolescence where hip function is vulnerable, so that health systems can plan for surveillance and detection. Therefore, the primary purpose of this scoping review was to identify, describe, and synthesize the available evidence on the longitudinal relationship between hip displacement and hip function in children and adolescents with CP up to the age of 18 years. Specific objectives were to: (1) describe the research designs, characteristics of the study populations, and definitions used to determine hip displacement and hip function; (2) categorize the measures of hip function used, in accordance with the ICF framework; and (3) chart the findings of studies that longitudinally reported hip displacement and hip function.

## METHOD

A scoping review was deemed suitable because of the broad and heterogenous nature of the research question. The Arksey and O'Malley scoping review framework,^27^ which consists of five phases, was used to guide the review: (1) identify the research question; (2) search for relevant studies; (3) select studies; (4) chart the data; and (5) collate, summarize, and report the results. The review protocol was registered prospectively with the Open Science Framework (https://osf.io/wymhq/). Reporting of the review followed the Preferred Reporting Items for Systematic reviews and Meta‐Analyses extension for Scoping Reviews (PRISMA‐ScR).^28^

### Identifying the research question

The Population, Concept, Context framework^29^ from the JBI was used to generate the research questions. Population was defined as children under the age of 18 years with a diagnosis of CP. Concept included hip displacement and hip function. Hip displacement refers to lateral displacement of the femoral head in the acetabulum of the pelvis, which is determined radiologically.^30^ The ICF core sets for children and youth with CP^31^ were used to provide a broad context for hip function and included items related to body functions and activities, and participations functions (Table S1). Context was framed as longitudinal studies where data were collected at least on two separate occasions to ascertain radiographic changes in hip structure and hip function over time.

### Study eligibility

We included studies with participants who were children or adolescents aged younger than 18 years at the time of study entry and who had a diagnosis of CP. Hip displacement must have been confirmed radiologically. Outcomes must have included any measure of hip function as defined by the ICF core set domains, such as hip pain, hip range of motion (ROM), hip muscle strength, gait, or hip‐related functional activity. Study designs could have included prospective, retrospective, or intervention studies, where radiological and functional data were collected for a minimum of two time points. Studies that were published as case studies or case series with fewer than 10 participants and research published only as an abstract, with no full‐text available, or as a protocol were excluded.

### Search strategy

The search strategy was developed with the assistance of a medical librarian, using the three‐step search strategy process recommended by the JBI.^32^ We identified key words and index terms from the title and abstract of relevant articles. The following electronic databases were searched from inception to May 2022 using these terms, categorized under population, concept, and context: MEDLINE (via EBSCO); EMBASE (via Elsevier); CINAHL (via EBSCO); and PsycINFO. The search strategy used is shown in Appendix S1.

All citations identified from the searches were imported into EndNote X9 (Clarivate, Philadelphia, PA, USA) and duplicates were removed. Citations were imported into the Covidence screening and data extraction software (Veritas Health Innovation, Melbourne, Australia). Titles and abstracts were initially screened independently by two reviewers (GT and HPF) in accordance with our eligibility criteria. Where studies met the eligibility criteria or eligibility was unclear, full texts were retrieved and assessed independently by two reviewers (GT and AM). Full texts not in English were machine‐translated using Google Translate to facilitate assessment for eligibility. Where disagreements arose, studies were discussed with a third reviewer (AM or HPF).

### Data charting

One reviewer (GT) extracted all relevant data from each study included, which were then reviewed by the other authors (HPF and AM) using a standardized form created in Microsoft Excel. The following data were extracted: country, setting, study design, inclusion and exclusion criteria, number of study participants, number of eligible participants and eligible hips, participant age range and sex, details of any interventions provided, number of measurement time points, follow‐up period, measure(s) of hip function, ICF domains assessed, receipt of research ethics approval, description of hip displacement, and study results. In line with JBI guidance and PRISMA‐ScR guidelines, risk of bias assessment was not undertaken.^29^

### Analysis and presentation of results

Data were collated from each study and tabulated, with a descriptive analysis of all studies included, which was completed according to the aims and objectives of the review. Limitations of the review and recommendations for future research were outlined to guide future research directions.

## RESULTS

A total of 1017 studies were identified after searching across the databases. After removal of 458 duplicates, 559 studies were screened according to title and abstract, 102 of which were retained for full‐text screening. After full‐text screening, 29 studies were included in this review. Four of 29 studies were not in English and were included after assessment using machine translation (Google Translate). The PRISMA‐ScR flow diagram is shown in Figure S1.

### Objective 1: Describe the research design, characteristics of the study populations, and definitions used to determine hip displacement and hip function

#### Characteristics of the studies included

Table 1 reports the main characteristics of the studies included. Studies were published in 16 countries; three in each of Australia,^33^, ^34^, ^35^ South Korea,^36^, ^37^, ^38^ Sweden,^5^, ^39^, ^40^ and USA;^41^, ^42^, ^43^ two in each of Canada,^44^, ^45^ Norway,^46^, ^47^ UK,^48^, ^49^ and Germany;^50^, ^51^ and one each in Austria,^52^ Egypt,^53^ France,^54^ Czech Republic,^55^ Serbia,^56^ Turkey,^57^ Brazil,^58^ and Spain.^59^ Publication year ranged from 1995 to 2021, with 12 (41%) published in the last 10 years (since 2014), and six (21%) published in the last 5 years (since 2019).

**TABLE 1:** Characteristics of the studies included in the review.

Study	Country	Setting	Study design	Total number of participants	Number of relevant participants	GMFCS level	Age	Intervention	Follow‐up period
Abdo et al.^58^	Brazil	Orthopaedic	Retrospective	17	17	IV and V	Mean = 6.8 years (range = 2.34–13.25 years)	Surgery	Mean = 6.1 years (range: 2.5–12.5 years)
Al‐Ghadir et al.^60^	Canada	Orthopaedic	Retrospective	39	39	IV and V	Mean = 8.1 years (range = 4.5–11.7 years)	Surgery	Median = 53 months (IQR = 24.9 months)
Aly et al.^53^	Egypt	Orthopaedic	Prospective case‐series	26 (11 female, 15 male)	26	IV and V	Range = 4.0–14.0 years	Surgery	Mean = 36.8 months (range = 2–4 years)
Atar et al.^41^	USA	Orthopaedic	Retrospective	36 (14 female, 12 male)	36	Not recorded	Range = 2–15 years	Surgery	Mean = 3.8 years (range = 3–6 years)
Bertoncelli et al.^54^	France	Surveillance	Longitudinal, retrospective, multicentre, double‐blinded	102 (42 female, 60 male)	102	III–V	Range = 15.3–17.7 years	BoNT‐A injection; hip surgery	Once a year for 12 years
Boyd et al.^33^	Australia	CP clinics	RCT	39 (15 female, 24 male)	39	II–V	Range = 1.6–4.8 years	BoNT‐A injection	1 year
Cho et al.^36^	South Korea	Rehabilitation	Retrospective	57 (26 female, 31 male)	57	I–V	Range = 2.0–6.0 years	BoNT‐A injection	Range = 5–10 years
Cobanoglu et al.^57^	Turkey	Orthopaedic	Retrospective	30 (14 female, 16 male)	30	I–V	Range = 5.0–18.0 years	Surgery	Mean = 57 months (range = 2–11 years)
Cobeljic et al.^56^	Serbia	Orthopaedic	Retrospective	42 (21 female, 21 male)	42	I–III	Range = 2.0–10.0 years	Surgery	Range = 3–18 years
DiFazio et al.^42^	USA	CP clinics	Prospective	38 (15 female, 23 male)	38	IV and V	Mean = 10.1 years (SD = 3.90)	Surgery	6 weeks and 3, 6, 12, and 24 months postoperatively
Hagglund et al.^5^	Sweden	Surveillance	Longitudinal registry study	212 (sex not recorded)	212	I–V	Range = 2.0–9.0 years	None	9 to 16 years. Two follow‐ups per year, age 2–6 years, one per year thereafter
Khot et al.^34^	Australia	Combined surgical and medical	Prospective cohort pilot study	16 (7 female, 9 male)	16	III and IV	Range = 2.0–6.0 years	Surgery; BoNT‐A injection	2 years after surgery (ROM every 3 months, X‐rays every 12 months)
Krebs et al.^52^	Austria	Orthopaedic	Retrospective	51 (sex not recorded)	51	I–V	Mean = 8 years 6 months (range = 1 year 8 months–19 years 7 months)	Surgery	Mean = 4 years, 10 months, (range = 11 months–11 years 10 months)
Larsen et al.^46^	Norway	Surveillance	Registry	67 (28 female, 39 male)	67	IV and V	Mean age = 14 years 7 months (SD = 1 year 5 months); range = 12–17 years	None	Range = 3 years 8 months–5 years 11 months
Lee et al.^38^	South Korea	Rehabilitation	Prospective	20 (6 female, 14 male)	20	IV and V	Mean = 5 years 1 months (SD = 1 year, 10 months); range = 2.0–10.0 years	BoNT‐A injection	1, 2, 3, 7, and 12 months
Martinsson and Himmelmann^39^	Sweden	Rehabilitation	Prospective case‐series	97 (48 female, 49 male)	97	III–V	Range = 2.0–6.0 years	Straddled standing; surgery	1 year
Martinsson and Himmelmann^40^	Sweden	Surveillance (longitudinal, retrospective, case–control study)	Rehabilitation	269 (118 female, 151 male)	269	IV and V	Median age = 3.7 years; range = 0.6–16 years	Stander or standing shell	Median = 1.5 years (range = 0.8–7.5 years) from baseline; median = 3.5 years (range = 0.5–8.7 years) from intervention
Moreau et al.^45^	Canada	Orthopaedic	Prospective	22 (10 female, 12 male)	22	IV and V	Mean = 4 years; range = 2.0–6.0 years	Surgery	Yearly for radiographs and videos; at 2 years and 5 years for questionnaires
Park et al.^37^	South Korea	Surveillance	Retrospective	48	48	III–V	Mean = 4.5 years	Study group: obturator nerve block; control group: no block or BoNT‐A injection	Mean (SD) duration of follow‐up: control group 20.10 months (5.60); study group: 18.46 months (6.50)
Pountney et al.^48^	UK	Rehabilitation	Prospective cohort study	39 (16 female, 23 male)	39	III–V	Not reported. All younger than 1.5 years at enrolment. Mean age at the 30‐month follow‐up = 2.6 years. Mean age at the 5‐year follow‐up = 5.1 years	24‐hour Chailey postural management	30 months and 60 months
Pountney et al.^49^	UK	Rehabilitation	Retrospective	59	59	Not recorded	(range = 5 months–9.8 years)	24‐hour postural management	Range = 1.2–16.9 years
Rodríguez‐Piñero Durán et al.^59^	Spain	Orthopaedic	Prospective	10 (4 female)	10	III–V	Mean = 5.5 years (SD = 2.27 years)	BoNT‐A injection	1‐, 3‐ and 6‐months post BoNT‐A injection
Rolauffs et al.^50^	Germany	Orthopaedic	Prospective	91 (46 female)	41	NR	Mean = 4 years 11 months (range = 1.1–15.8 years)	Physiotherapy, surgery	1, 2, 3, and 4 years
Rolauffs et al.^51^	Germany	Orthopaedic	Prospective	91 (46 female)	91	NR	Mean = 4.9 years (range = 1.1–15.8 years)	Surgery	1, 2, 3, and 4 years
Roposch and Wedge^44^	Canada	Orthopaedic	Retrospective	32 (14 female)	32	IV and V	Range = 5.2–16.8 years	Surgery	Mean = 5.3 years (range = 2–11.7 years)
Rutz et al.^35^	Australia	Orthopaedic	Retrospective	11 (5 female, 6 male)	11	I and II	Range = 7–16 years	Surgery	Range = 2 years 3 months–10 years 8 months
Schejbalova et al.^55^	Czech Republic	Orthopaedic	Retrospective	35 (sex not recorded)	35	IV and V	Range = 9–18 years	Surgery	Radiography at 3, 6, and 12 months; clinical follow‐up yearly. Mean follow‐up at 98 ± 4.5 months
Silverio et al.^43^	USA	Orthopaedic	Retrospective	12 (3 female, 9 male)	12	V	Range = 9–18 years	Surgery	Range = 24–60 months
Terjesen^47^	Norway	Surveillance	Prospective	31 (11 female, 20 male)	31	IV and V	Range = 2.2–9.9 years	Surgery	Range = 3.8–11 years

Ages are reported in decimal years as in the original studies. Abbreviations: BoNT‐A, botulinum neurotoxin A; IQR, interquartile range; RCT, randomized controlled trial.

A range of study designs were used, including four longitudinal registry‐based studies from 2007 to 2021, one from Norway^46^ and three from Sweden;^5^, ^39^, ^40^ one randomized controlled trial;^33^ 12 prospective studies;^34^, ^38^, ^39^, ^42^, ^45^, ^47^, ^48^, ^50^, ^51^, ^53^, ^54^, ^59^ and 12 retrospective designs from 1995 to 2018.^35^, ^36^, ^37^, ^41^, ^43^, ^44^, ^49^, ^55^, ^56^, ^57^, ^58^, ^60^

Studies were conducted in several settings, including orthopaedic clinics,^41^, ^43^, ^45^, ^50^, ^51^, ^52^, ^53^, ^56^, ^57^, ^58^, ^59^, ^60^, ^61^ rehabilitation settings,^36^, ^38^, ^39^, ^40^, ^48^, ^49^ population‐based registries,^5^, ^40^, ^46^, ^54^ or CP clinics.^33^, ^42^ The setting of one study, which evaluated a combined medical and surgical intervention, was not specified.^34^

Fifteen (52%) of the studies reported receiving research ethics approval.^33^, ^35^, ^36^, ^37^, ^42^, ^43^, ^44^, ^46^, ^47^, ^49^, ^54^, ^56^, ^57^, ^58^, ^60^

#### Assessment time points

Follow‐up assessment varied across the studies from a minimum of 1 year^39^ to a maximum of 18 years.^56^ Assessment time points were standardized in one registry study,^5^ seven prospective studies,^42^, ^47^, ^48^, ^50^, ^51^, ^57^, ^59^ one randomized controlled trial,^33^ and one retrospective study that reported conducting radiography assessments at 3‐month, 6‐month, and 12‐month intervals, and clinical assessments annually.^55^ A second retrospective study collected data from electronic health records based on an annual assessment over a 12‐year period.^54^ It was not clear from the remaining prospective studies if the follow‐up period was standardized.^38^, ^39^, ^45^, ^53^ Because of the nature of data collection (predominantly from medical records), follow‐up periods were variable in the remaining retrospective studies.^40^, ^41^, ^43^, ^52^, ^56^, ^60^ Follow‐up time points were usually reported as a range (minimum to maximum) and mean (Table 1).

#### Participants

The 29 studies report on a total of 1548 participants. Two of the studies^43^, ^44^ involved an overlapping cohort of participants; while these studies were eligible separately as they reported different outcomes, we counted their participants just once for this total. Sample sizes ranged from 11^35^ to 267.^40^ Sex was reported by 25 studies, in which 43% were females. Using an age classification of preschool (≤6 years), primary school (7–12 years), and adolescence (13–19 years), seven studies^33^, ^34^, ^36^, ^37^, ^39^, ^45^, ^48^ included only preschool children at baseline assessment. A further seven studies^5^, ^38^, ^47^, ^49^, ^53^, ^56^, ^60^ included a mix of preschool and primary school children. Two studies^46^, ^54^ included adolescents only. The remaining 13 studies^35^, ^40^, ^41^, ^42^, ^43^, ^44^, ^50^, ^51^, ^52^, ^55^, ^57^, ^58^, ^59^ included children across a mixed range of ages from preschool to adolescence. The youngest participant was 5 months old at study commencement.^49^ Twenty‐three studies recruited participants under the age of 6 years.

Sixteen studies specified the eligibility criteria for GMFCS level or ambulatory status. Ten studies included non‐ambulatory children classified in GMFCS level IV or V only.^38^, ^40^, ^42^, ^44^, ^45^, ^46^, ^53^, ^55^, ^58^, ^60^ A further four studies reported outcomes for a mixed cohort of ambulatory and non‐ambulatory children classified in GMFCS levels II to V.^34^, ^37^, ^39^, ^59^ In two studies, all participants were ambulatory (GMFCS levels I–III).^35^, ^56^ The remaining 11 studies did not prespecify eligibility with regard to ambulation or GMFCS level.

Sixteen studies specified eligibility criteria for CP subtype or distribution. Children with spastic bilateral CP were eligible for inclusion in nine studies.^33^, ^37^, ^38^, ^42^, ^45^, ^46^, ^52^, ^56^, ^60^ A further two studies included children with mixed CP subtypes.^53^, ^54^ Five studies specified the eligibility criteria for CP distribution; of these, three included children with bilateral CP^46^, ^48^, ^49^ and one included children with unilateral CP.^35^

#### Measurement of hip displacement

To be eligible for inclusion in this review, hip displacement must have been confirmed radiologically. A range of radiographic measures were used, with RMP used most commonly,^5^, ^33^, ^34^, ^35^, ^36^, ^37^, ^38^, ^39^, ^40^, ^42^, ^44^, ^46^, ^47^, ^48^, ^49^, ^50^, ^51^, ^53^, ^54^, ^56^, ^58^, ^59^, ^60^ as outlined in Table S2. The acetabular index (Sharp's angle) was used in four studies.^35^, ^53^, ^58^, ^60^ The centre‐edge angle of the pelvis was measured in four studies,^35^, ^45^, ^52^, ^60^ while the neck‐shaft angle of the femur was measured in two studies.^41^, ^60^ One study, which included children with irreducible hip dislocation, verified using clinical and radiological examination, did not report the radiographic measure used.^55^ One study measured the presence and severity of heterotrophic ossification, as well as prosthesis migration, in children undergoing proximal femur prosthetic interposition arthroplasty.^43^ The severity of hip displacement at study commencement varied across studies. RMP was an inclusion criterion for some studies, using more than 30%,^35^ more than 33%,^53^, ^56^ 10% to 40%,^33^ 25% to 45%,^34^ or 20% to 60%.^37^ Nine studies, which evaluated the effect of surgery, used an inclusion criterion of subluxation or dislocation at study commencement, rather than specific measures of hip displacement.^41^, ^43^, ^44^, ^45^, ^47^, ^52^, ^55^, ^57^, ^60^ As most of these were retrospective, only two provided baseline (preoperative) data on the severity of hip displacement.^45^, ^47^

#### Change in radiographic measures over time

Changes in hip displacement measured radiographically were reported differently across studies. A change in RMP expressed as a mean or median was reported in 16 studies,^35^, ^37^, ^39^, ^40^, ^41^, ^42^, ^44^, ^46^, ^47^, ^50^, ^51^, ^53^, ^56^, ^58^, ^59^, ^60^ the progression rate of RMP in two studies,^38^, ^47^ and change in the number or proportion of hips in the different migration percentage categories in two studies.^33^, ^48^ A change in mean centre‐edge angle was reported in two studies,^52^, ^60^ a change in acetabular index in three studies,^52^, ^53^, ^60^ a change in femoral neck‐shaft angle in two studies,^41^, ^60^ the number of subluxed and dislocated hips in two studies,^48^, ^57^ and the number in a safe RMP range (<33% migrated)^49^ in one study; one study provided a subjective report of ‘correct X‐ray appearance’.^55^

### Objective 2: Categorize the measures of hip function used in accordance with the ICF framework

#### ICF components

Twenty‐six studies (90%) reported outcomes at the body structure and function impairment level of the ICF. The most frequently measured outcome was range of movement (ICF code B710, mobility of joint functions, 16 studies).^5^, ^33^, ^34^, ^36^, ^38^, ^39^, ^40^, ^43^, ^50^, ^51^, ^53^, ^54^, ^55^, ^58^, ^59^, ^60^ This was followed by hip pain (ICF code B280, sensation of pain, nine studies)^43^, ^44^, ^45^, ^46^, ^47^, ^52^, ^55^, ^57^, ^60^ and spasticity (ICF code B755, involuntary movement reaction functions, five studies, measured using either the Modified Ashworth Scale or Tardieu Scale).^33^, ^36^, ^37^, ^38^, ^59^ One study used three‐dimensional gait analysis and reported the Gait Profile Score and Movement Analysis Profile (ICF code B77, gait pattern functions).^35^

Seventeen studies (59%) measured change in the activity component of the ICF, namely codes D455 (moving around), D420 (transferring oneself), and D415 (maintaining a body position). Three studies reported the Functional Mobility Scale,^34^, ^54^, ^56^ two studies reported Chailey levels of ability,^48^, ^49^ one study reported the Gross Motor Function Measure,^33^ a further study reported the Trunk Impairment Scale, Lower Extremity Functional Scale, Posture and Postural Ability Scale, and modified Harris Hip Score,^54^ two studies reported the Rancho Los Amigos classification of psychomotor abilities,^50^, ^51^ and one study reported the Barthel index.^52^ Six studies assessed activity limitation according to parent or caregiver report, using non‐standardized ordinal scales or custom questionnaires.^41^, ^43^, ^44^, ^45^, ^57^, ^60^ Three studies^38^, ^42^, ^53^ reported the Caregiver Priorities and Child Health Index of Life with Disabilities (CPCHILD), a measure that spans the activity and participation dimensions of the ICF. No other participation measures were reported. Further details are provided in Table S2.

### Objective 3: Charting the findings of studies that reported hip displacement and hip function longitudinally

#### Natural history

Two studies included in the review, both longitudinal, did not include interventions, thereby providing findings indicative of natural history. One prospective longitudinal study^5^ reported on 212 children from the CPUP Register in Sweden who had a baseline assessment (consisting of hip ROM and radiography) between the ages of 2 years and 9 years, and were followed up at least annually for a further 7 years. During this time, 27% of children developed an RMP greater than 33% (9%, RMP 33%–50%; 18%, RMP >40%). No child developed hip dislocation. The most common age at first registration of an RMP greater than 33% was 3 to 4 years. Hip displacement was associated with GMFCS but not with the primary functional outcome of hip ROM.

Another prospective study^46^ reported on 67 adolescents, aged 12 to 17 years, assessing pain and function via telephone interview and hip radiography over a 5‐year follow‐up. The prevalence of pain increased from 18 of 67 (27%) participants at baseline to 28 of 67 (42%) participants at the follow‐up, while the mean RMP of the most affected hip was unchanged. Hip pain was more prevalent in GMFCS level V and adolescents with severe subluxation respectively. Ten participants underwent hip surgery during the 5‐year period.

#### Interventions

Twenty‐seven of the 29 studies entailed delivery of an intervention, with measurements taken before and after the intervention, at several follow‐ups. Interventions included surgery to address bony abnormalities and soft‐tissue changes, physiotherapy, and nerve block or botulinum neurotoxin A (BoNT‐A) injections. Surgery to address bony abnormalities included different types of femoral or pelvic osteotomy, or proximal femoral arthroplasty.^35^, ^41^, ^42^, ^43^, ^44^, ^47^, ^53^, ^55^, ^57^, ^58^, ^60^ A range of soft‐tissue lengthening procedures included tenotomies and tendon releases of various hip muscles.^5^, ^39^, ^45^, ^47^, ^50^, ^51^, ^54^, ^56^, ^57^

A variety of soft‐tissue procedures and osteotomies were undertaken in seven studies;^35^, ^41^, ^42^, ^47^, ^52^, ^53^, ^57^ a combination of conservative and surgical procedures was provided in three studies.^34^, ^35^, ^54^

Physiotherapy included postural management^48^, ^49^ and standing frames,^39^, ^40^ while non‐surgical spasticity management interventions included nerve block^34^, ^37^ and BoNT‐A injections.^33^, ^36^, ^38^, ^54^, ^59^ More detail of the interventions is provided in Table S3.

#### Age at intervention

**FIGURE 1:**
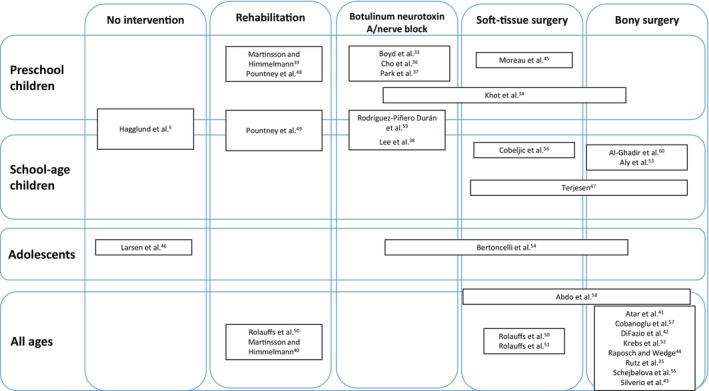
Schematic map of interventions reported in each study and the ages of the study participants at baseline. Where a study included participants across two age groups, this is denoted by the study identifier crossing both groups.

Figure 1 shows a schematic map of interventions reported across the studies and the ages at baseline of the study participants. Thirteen studies reported outcomes on children who had an intervention across all ages, of which 12 reported surgical interventions;^35^, ^41^, ^42^, ^43^, ^44^, ^50^, ^51^, ^52^, ^55^, ^57^, ^58^ and one study reported a rehabilitation intervention.^40^ Seven studies reported the outcomes of interventions where children were of preschool age at enrolment, three of which reported outcomes after BoNT‐A injections or nerve block,^33^, ^36^, ^37^ two reported outcomes after a rehabilitation intervention,^39^, ^48^ one reported soft‐tissue surgery,^45^ and one reported several interventions, including BoNT‐A, soft‐tissue surgery, and bony surgery.^34^ Two studies, one of participants who had a rehabilitation intervention^49^ and one of participants who had BoNT‐A injections,^38^ enrolled participants across preschool and school ages. Four studies reported on school‐age children; two reported bony surgery^53^, ^60^ and two reported soft‐tissue surgery. One study reported the outcomes of the intervention (BoNT‐A, soft‐tissue or bony surgery) at adolescence.^54^ Of the two longitudinal studies that did not specifically report on an intervention, one was of children at preschool and school age,^5^ and the other of adolescents.^46^

#### Follow‐up

The follow‐up period varied across the studies from an overall minimum of 1 month to a maximum of 18 years.^56^ Thirteen studies (10 prospective studies,^5^, ^34^, ^38^, ^39^, ^42^, ^45^, ^50^, ^51^, ^55^, ^59^ one randomized controlled trial,^33^ and two retrospective studies^54^, ^55^) reported follow‐up data at prespecified intervals, most commonly 1 year.^33^, ^38^, ^39^, ^42^, ^54^, ^55^ Some included shorter follow‐ups of 1 month^38^, ^42^ 3 months,^38^, ^42^, ^55^ or 6 to 7 months.^5^, ^38^, ^42^, ^55^ Khot et al.^34^ and DiFazio et al.^42^ followed up for 24 months and Rolauffs et al.^50^, ^51^ for 48 months. Bertoncelli et al.^54^ reported annual follow‐ups for a minimum of 3 years and a maximum of 12 years. The remaining 17 studies reported variable follow‐up intervals ranging from 10 months^40^ to 18 years.^56^

#### Changes in function over time and relationship with hip displacement

Exploring the relationship between hip ROM and radiology, Cho et al.,^36^ in their retrospective study of 57 participants, reported an inverse correlation between RMP and range of hip abduction and hip flexion, but no relationship between spasticity and femoral anteversion angle, whereas Hagglund et al.^5^ (prospective registry study, *n* = 212) found no correlation between ROM and radiological measures. Other studies reported changes in ROM alongside radiological changes but did not explore these for evidence of association. Specifically, Aly et al.^53^ and Al‐Ghadir et al.^60^ found improved hip abduction of 19 to 23 degrees after bony surgery, coinciding with a reduction in RMP, Mckibbin index, and acetabular index, although they did not test for strength of correlation. Aly et al.^53^ found a reduction in hip flexion contracture from 38.6 to 1.4 degrees, coinciding with improved radiological measures after surgery. Regarding a change in ROM after non‐surgical interventions, in a single‐arm intervention study of BoNT‐A injections to the hip adductor muscles of 20 children aged 2 to 10 years, Lee et al.^38^ found increases in hip abduction range from a mean of 29 degrees to a mean of 45 degrees over 12 months, with the RMP remaining stable (0.4% change). After BoNT‐A injections to the hip flexors, Rodríguez‐Piñero Durán et al.^59^ reported significant improvements in hip flexion ROM from baseline to 6 months coinciding with a decrease in RMP, although the follow‐up was incomplete. For rehabilitation interventions, Martinsson and Himmelmann^39^, ^40^ found improvements in hip abduction ROM (measured categorically) and RMP in children who undertook at least 1 hour per day of straddled standing, but did not explore evidence of association. Lee et al.^38^ and Park et al.^37^ reported improvements in spasticity (measured with the Modified Ashworth Scale) after BoNT‐A and obturator nerve block interventions respectively, with minimal or no changes in radiology.

Measuring pain over a follow‐up period of 5 years, Larsen et al.^46^ found that the number of adolescents with hip pain increased from 18 to 28 of 67 participants, while the mean RMP of the most displaced hip was unchanged.

The single study reporting three‐dimensional gait outcomes^35^ found improvements after multilevel orthopaedic surgery overall in Gait Profile Score and segmentally in hip and pelvic rotation (measured using the Movement Analysis Profile) at the 1‐year follow‐up. The study did not explore for evidence of association between these changes and the small radiological improvement in RMP. The study methods describe a long‐term follow‐up at 4 to 5 years, but gait outcomes are not reported at this longer follow‐up.

Two studies provide evidence of change in activity measures alongside radiology. Atar et al.^41^ found maintenance of sitting and ambulation performance after varus derotation osteotomy, but this did not correlate with improved radiology. DiFazio et al.^42^ described trends of change in radiology and a mixed activity and participation measure (CPCHILD). They found that preoperative RMP was negatively correlated with preoperative CPCHILD. Postoperatively, both RMP and CPCHILD improved, but improvement in RMP occurred within 6 weeks, during which time the CPCHILD scores declined, with subsequent slow recovery of scores over 12 months.

## DISCUSSION

This scoping review aimed to identify, describe, and synthesize the available evidence on the longitudinal relationship between hip displacement and hip function in children and adolescents with CP up to the age of 18 years. Overall, we found that the relationship between hip structure (displacement) and function (particularly at the activity and participation levels of the ICF) from infancy to adolescence is poorly understood for several reasons. First, most evidence focuses on the outcomes of interventions, primarily surgery, and also BoNT‐A or nerve block interventions. Most studies reported improvements in radiological and functional outcomes after the intervention; however, missing from this trajectory is the picture of hip function that preceded deterioration before the intervention. We found gaps in the monitoring of children who had no intervention, although notably, two registry studies contributed knowledge in this regard. Second, intervention studies were mostly retrospective in design. While we did not undertake quality assessment, it is clear that retrospective data extraction will be problematic in the quality and consistency of outcome measurement unless strict protocols are in place. Third, measurement of hip function was mostly limited to impairment measures of ROM or pain, with little representation of activity and minimal measurement of participation. Therefore, we found significant gaps in the evidence base on the longitudinal relationship between hip structure and function.

Many radiological measures of hip geometry have been used to evaluate hip displacement. The most commonly used measure was RMP, which was first described in 1980.^13^ A direct relationship has been established between an RMP of 30% to 34% and GMFCS levels.^5^, ^6^ RMP cut‐off values of 33% and 90% are recommended for subluxation and dislocation respectively.^13^ While a change in RMP greater than 10% is clinically important,^62^, ^63^ the initial degree of hip displacement needs to be considered in the context of change over time and impact on function. In this review, several radiological measures were used to define hip displacement, with varying degrees of severity up to complete dislocation. Interpreting and comparing changes in hip displacement longitudinally and how they relate to hip‐related function is difficult because of the variety of methods used to measure hip displacement, particularly in the studies that described changes categorically, rather than according to mean or percentage change.

Interpretation of radiology also depends on psychometric properties. A systematic review of 19 studies, which aimed to ascertain the reliability and validity of radiological methods to assess hip geometry in children with CP, found that RMP has good concurrent validity and good to excellent intrarater and interrater reliability, with improvement of reliability with increasing age and femoral head size.^30^ Neck‐shaft angle and acetabular index also had good to excellent reliability and validity and were suggested by the authors to be the criterion standard for diagnosis in hip surveillance.^30^ Our review found inconsistent reporting of these measures. Neck‐shaft and head‐shaft angles measure femoral valgus deformity, with neck‐shaft angle more correlated to migration percentage than head‐shaft angle.^64^ RMP and head‐shaft angle were prioritized in a recent consensus on a core measurement set for radiological assessment of hip disease in CP, which has potential to improve future reporting.^65^ However, while several radiological measures were reported in this review, many are not used routinely. A recent Delphi consensus study identified two radiographic measurements, RMP and head‐shaft angle, to be included in a core measurement set for clinical practice, research, and surveillance programmes.^65^

The interventions in the studies were undertaken based on individual clinical need, most probably after hip radiology or once hip function had declined to a significant extent.^47^ Thus, the evidence base gives limited understanding of what happened before the intervention (particularly bony surgery) was deemed necessary. To fully understand the relationship between hip structure and hip function, it is necessary to explore change over time with the hip in its ‘natural’ (before surgery) alignment. In Ireland, a longitudinal study of three‐dimensional gait analysis in an untreated cohort of 180 children with CP showed a tendency to reduced hip extension and abduction during gait over a mean 4‐year 11‐months follow‐up from childhood to adolescence. Interestingly, this study found no difference in age‐related progression of gait outcomes between children who had received a recommendation for surgical intervention (but chose not to), and children who had never been recommended surgery.^66^ The authors recommend that treatment can only be deemed successful if outcomes at least match typical age‐related changes.

However, interpretation of success further depends on which outcomes are measured. Our review is limited in drawing conclusions about the relationship between hip structure and function over time largely because of the limited representation of function by the outcome measures reported in the studies. Hip ROM was the most commonly studied outcome, but findings differed as to whether hip ROM over time was associated with changes in radiological measures, and it is problematically variable in non‐ambulatory children who are most at risk of hip displacement.^15^ Two studies^53^, ^60^ reported significantly improved hip ROM and reduced contracture alongside postoperative improvements in radiological measures of displacement, whereas a registry study^5^ did not find an association between changes in radiology and ROM over time. Similarly, Larsen et al.^46^ found no evidence of a longitudinal relationship between radiological measures of hip structure and pain, despite increasing pain prevalence over the 5‐year follow‐up. Our review found underrepresentation of activity‐level outcomes and sparse reporting of participation, limited to the CPCHILD (reported by three studies), which is predominantly an activity‐level measure but includes three questions on participation. As no study used a pure or predominantly participation‐level measure, it is not known how hip structure might affect participation over time. This is an important gap because it is long understood that the relationship between body structure and function, and activity and participation, is far from linear. There is a need for future studies to consider outcomes across the ICF, particularly when reporting outcomes from invasive interventions, where there is an initial deleterious effect on participation.^42^

A feature of this scoping review was the dearth of rehabilitation interventions reported by the studies included in the review. The goals of rehabilitation typically relate to activity or participation, and interventions are not usually designed with the intent to influence a radiological measure. This may explain the low representation of studies with rehabilitation interventions within our eligibility criteria. The findings of Martinsson and Himmelmann^39^, ^40^ suggest that at least 1 hour per day of straddled (abduction) standing, whether as a standalone intervention or after surgery, may be associated with improved gains in hip abduction ROM and reduced RMP. A prospective cohort study of 39 children, enrolled while under 18 months of age,^48^ concluded that early provision of postural management equipment helps reduce the number of hip problems at 5 years of age. From a methodological quality perspective, the certainty of this conclusion is low because the control group was historical, the primary outcome (Chailey levels of ability) is an ordinal scale with few categories making it insensitive to change, and the study did not record other interventions.

Our review further underpins the rich contribution of registry studies. Recognizing the significant impact of hip displacement on morbidity in CP, and in an attempt to reduce the rate of hip dislocation, hip surveillance programmes were established internationally with the launch of the first programme (CPUP) in Sweden in 1994.^15^ The main goal of hip surveillance is to provide systematic hip screening to prevent complete hip dislocation.^6^ While the details of each surveillance schedule vary, each programme incorporates radiological and clinical evaluation.^61^ Surveillance programmes have significantly reduced the rate of hip dislocation and corrective surgeries required.^67^ Regarding the outcomes that should be captured to address some of the gaps we identified in this review, core outcome sets are useful and have been established for CP in children and young people,^31^ and in adults; however, these provide guidance on ‘what’ to measure but do not specify ‘how’ it should be done (with which measures). Selection of functional measures needs to balance functional goals with the feasibility of surveillance at the population level and the many competing factors as summarized by Oeffinger.^68^ Children functioning in GMFCS levels IV and V, who are non‐ambulatory, are at higher risk of hip displacement and need particular focus in registry data for hip surveillance. In their protocol for the Netherlands CP register, Andringa et al.^69^ describe an approach to collecting longitudinal data on surveillance and treatment outcomes, including clinical outcomes and patient‐reported and parent‐reported outcomes across ICF domains. Their dashboard presents CPCHILD in its component scores, enabling functions relevant to the non‐ambulatory child (personal care; sitting, standing, and moving) to be observed over time, and includes the Goal Attainment Scale for individual outcomes.

### Strengths and limitations of the review

The findings of our review are strengthened by its methodological rigour, including the use of a rigorous scoping review framework,^27^ comprehensive search strategies across multiple databases, and mapping of the functional outcomes to the ICF framework. This review has some limitations. First, the studies included were significantly heterogeneous in study design, population (inclusion criteria, age and functional ability of participants), intervention, outcome measurement, and follow‐up. This makes evidence synthesis challenging. Second, our criterion of two radiological measurements at baseline and follow‐up led to the exclusion of some studies that included potentially valuable data on hip function; however, we deemed this criterion necessary to answer the question on the relationship between radiology and function over time. Finally, eight studies at the full‐text screening were published in languages other than English and we used machine translation (Google Translate) to determine eligibility and, for four that were eligible, to facilitate data extraction. Machine translation may have been imprecise.

### Recommendations for future research

Our findings lead us to several recommendations. First, there is a need for more longitudinal prospective studies with defined follow‐up intervals and core outcome sets, reported in compliance with guidelines such as STROBE (STrengthening the Reporting of OBservational studies in Epidemiology). The optimal follow‐up for registry studies is to be determined, but more frequent follow‐up at a younger age is prudent. Second, to overcome the predominance of intervention studies, there is a particular need for longitudinal data on a wider representative sample of children with CP. This would enable a more comprehensive understanding of natural history and could capture the reasons for deterioration that preceded the intervention, so that these factors can be understood and anticipated. Such data would also allow intervention studies, particularly those involving surgery, to be compared against natural history. Finally, outcome measurement should include activity‐level and particularly participation‐level outcomes to give a more holistic and person‐centred interpretation of both natural history and the effect of the intervention. Participation‐level outcome measurement should build in flexibility to include goals determined by the young person with CP and their family, in addition to standardized measurement.^69^ Participation outcomes can be lengthy to complete and some may not be feasible for registry data where 80% of data are entered by clinicians;^70^ however, the work of Mäenpää et al.^71^ provides a template for consensus. We recommend consultation with young people with CP and their families to inform this process.

To conclude, current evidence gives limited insight into the relationship between hip displacement (measured radiologically) and hip function, and how they change over time as children with CP grow and develop. The participation level of the ICF is particularly underrepresented in outcome measurement. Our findings support the need for prospective longitudinal data, especially in younger children and before major intervention.

## Supporting information


**Table S1:** ICF core set for children and youth with cerebral palsy items related to body functions, and activities and participation.

**Table S2:** Outcome measures for hip function (classified according to the ICF) and radiological measures of hip displacement.

**Table S3:** Study characteristics with detail of study interventions.

**Appendix S1:** MEDLINE search strategy.

**Figure S1:** Preferred Reporting Items for Systematic reviews and Meta‐Analyses extension for Scoping Reviews (PRISMA‐ScR) flow diagram.

## Data Availability

There are no additional data for this paper.
