# Supplementary material for: Longitudinal relationship between hip displacement and hip function in children and adolescents with cerebral palsy: A scoping review
Source: Dev Med Child Neurol. 2024 Nov 21;67(4):450–62. doi: 10.1111/dmcn.16175 (PMC11875528; doi:10.1111/dmcn.16175)
Supplement: Supplementary file 1 — Table S1: ICF core set for children and youth with cerebral palsy items related to body functions, and activities and participation. [file DMCN-67-450-s001.docx]

| **ICF Domain** | **Function** | **ICF Code** |
| --- | --- | --- |
| **Body Functions** | Sensation of pain | B280 |
|  | Mobility of joint functions | B710 |
|  | Stability of joint functions | B715 |
|  | Muscle power functions | B730 |
|  | Muscle endurance functions | B740 |
|  | Involuntary movement reaction functions | B755 |
|  | Control of voluntary movement functions | B760 |
|  | Gait pattern functions | B770 |
| **Activities and Participation Functions** | Changing basic body position | D410 |
|  | Maintaining a body position | D415 |
|  | Transferring oneself | D420 |
|  | Moving objects with lower extremities | D435 |
|  | Walking | D450 |
|  | Moving around | D455 |
|  | Moving around in different locations | D460 |
|  | Moving around using equipment | D465 |

**Table S1**

International Classification of Functioning of Functioning (ICF) core set^31^ for children and youth with cerebral palsy items related to body functions, and activities and participation.
