# Supplementary material for: Longitudinal relationship between hip displacement and hip function in children and adolescents with cerebral palsy: A scoping review
Source: Dev Med Child Neurol. 2024 Nov 21;67(4):450–62. doi: 10.1111/dmcn.16175 (PMC11875528; doi:10.1111/dmcn.16175)
Supplement: Supplementary file 2 — Table S2: Outcome measures for hip function (classified according to the ICF) and radiological measures of hip displacement. [file DMCN-67-450-s005.docx]

| **Author, year** | **Measure of Function (based on ICF)** | | | **Hip displacement parameter** | **Authors’ Conclusions** |  |
| --- | --- | --- | --- | --- | --- | --- |
|  | **Body Structure Function** | **Activity** | **Participation** |  |  |  |
| Abdo, 2016 | ROM |  |  | Acetabular angle, Reimer's MP, Shenton arc continuity (AC) | Prophylactic surgery on the contralateral hip is not justified as <50% proceed to subluxation on the contralateral side. Patients >8y old and MP <30% and AC <25% are at lower risk |  |
| Al-Ghadir, 2009 | Pain, ROM | Sitting comfort |  | 33-99% MP = subluxed, 100%= dislocated.  Centre edge angle, acetabular index, neck-shaft angle | Clinical and radiological results obtained by a one-stage surgical procedure (varus derotational osteotomy and San Diego osteotomy) were far better at an average follow-up time of 4.4. years than doing varus derotational osteotomy alone |  |
| Aly, 2019 | ROM | CPCHILD | CPCHILD | MP >33% and AI > 30° | Femoral varus derotation osteotomy and San-Diego pelvic osteotomy with soft tissue release on the affected side combined with varus derotation osteotomy with soft tissue release on the contralateral contained hip is beneficial to achieve hip containment and painless sitting balance in non-ambulatory children with CP. |  |
| Atar, 1995 |  | Walking and sitting scale |  | AI>40° was an indication for surgery due to dislocation or subluxation | Varus derotational osteotomy surgery combined with soft-tissue release and innominate osteotomy in a dysplastic hip was effective in maintaining hip reduction in patients with CP |  |
| Bertoncelli, 2021 | ROM | Trunk Impairment Scale  Functional Mobility Scale  Lower Extremity Functional Scale  Posture and Postural ability scale  Modified Harris Hip Score |  | <33% MP= normal; 33-89% MP= subluxed, ≥90% =dislocated, based on Perkins’ line | Trunk muscles tone disorder, severe scoliosis, epilepsy and spasticity were predictors of  neuromuscular hip dysplasia in teens with CP. |  |
| Boyd, 2001 | ROM, spasticity (Modified Ashworth Scale) | Gross motor function measure (GMFM) |  | MP between 10-40% | There was no treatment effect of  BTX-A and a variable hip abduction orthosis compared with current clinical care on GMFM at 12-months in children aged 1-4 years with CP |  |
| Cho, 2018 | ROM, spasticity (Modified Tardieu Scale) |  |  | MP based on Hilgenreiner’s line and Perkins line | Hip subluxation and coxa valga deformity correlated with both dynamic spasticity and shortening of hip adductor muscles. However, we found no correlation between femoral deformities such as femoral anteversion, coxa valga, and hip subluxation. |  |
| Cobanoglu, 2017 | Pain | Subjective report of difficulty with sitting, walking, perineal hygiene |  | Reimer's MP: 25-39%=mildly subluxed, 40-59% moderate, 60-99% severely, 100% dislocated | There is a positive relationship between GMFCS and hip displacement. There were no significant differences  between the preoperative and postoperative GMFCS levels  GMFCS levels stable after surgery. Relief in symptoms was not consistent with changes in GMFCS in children with CP after hip reconstruction |  |
| Cobeljic, 2009 |  | Functional mobility scale |  | MP >33%= Subluxed | Relative MP correction was superior in patients treated by rectus femoris and iliopsoas tenotomy with iliac crest resection (sartorius release)(group B) vs iliopsoas tenotomy (group A). No patient had MP progression in either the subloxed hip or non-subluxed hip. Walking ability improved in 55% of group A and 86% of group B patients |  |
| DiFazio, 2016 |  | CPCHILD | CPCHILD | MP >30% is a hip 'at risk' for subluxation, >50% is 'at risk' of dislocation | Preoperative migration percentage negatively correlated with the preoperative CPCHILD score and this relationship continued throughout follow-up ([post-surgery). There was no correlation between the acetabular index and CPCHILD total score before or after surgery | |
| Hagglund, 2007 | ROM |  |  | Subluxed between 33-40%. In hips with a "Gothic arch" formation of the lateral margin, the midpoint of the arch was used as reference point | Hip displacement often occurs by the age of 2-3. ROM is a poor indicator of hip at risk and cannot replace radiographic examinations for hip screening. However, a decreasing ROM over time in an individual child could warrant radiographic hip examination. | |
| Khot, 2008 | ROM | Functional mobility scale |  | Reimer’s MP | Combined surgical–medical intervention resulted in a reduction of spastic hip subluxation, and improvements in ROM and gross motor function. | |
| Krebs, 2008 | Pain | Barthel index |  | Wiberg’s Centre Edge angle, Cornell and Hilgrenreiner’s AI.  CE angle 20-0° =subluxation,  CE angle <0°= dislocation | Independence of patients based on the Barthel index did not change significantly after surgery. Most improvements in quality of life were observed in those who had hip pain pre-operatively, as a result of reduction of pain and improved hip mobility. | |
| Larsen, 2021 | Pain |  |  | Reimer’s MP: MP <33%=Normal, 33-89%=subluxed,  >90% = dislocated. Subluxation classified as mild 33-39%, moderate 40-49% and severe 50-89% | Over 5 years, the number of participants with hip pain increased from 18 to 28, while the mean migration percentage of the most displaced hip was unchanged. The CP hip surveillance programme did not protect participants against  increasing prevalence of hip pain during adolescence | |
| Lee, 2021 | ROM, spasticity (Modified Ashworth Scale) | CPCHILD | CPCHILD | Change in Reimer’s MP over 1 year as a measure of progression of hip displacement | Two sessions of botulinum toxin injections over 6 months at the hip adductor muscles significantly reduced muscle tone and hip displacement. A botulinum toxin injection may be used as an adjunctive treatment in prevention of hip dislocation.  . | |
| Martinsson, 2011 | ROM |  |  | MP categorised at <33%, 33-40% and >40% | Straddled weight bearing may reduce MP after adductor-iliopsoas tenotomies or prevent an MP increase and preserve muscle length in children with CP who did not need surgery. | |
| Martinsson, 2021 | ROM |  |  | MP | Median MP decreased 7.0% in the participants without surgery, increased 6.5% in their matched participant, and decreased 3.5% in those who had adductor-psoas tenotomy. MP decreased 18% and ROM increased in participants who performed abducted standing after surgery. Standing abduction at 15-30° for 10h/wk reduces MP and preserves ROM for up to 7 years | |
| Moreau, 1995 | Pain | Use of walking aids; ambulatory status; independence with ADLs |  | Centre Edge Angle | Adductor and psoas releases prevent hip subluxation and provide a measure of protection to opposite hip. There was an added benefit in functional improvement | |
| Park, 2014 | Modified Ashworth Scale, Modified Tardieu Scale |  |  | MP between 20-60% | MP and spasticity reduced in those who received Obturator nerve block at 6-months, whereas MP increased in the non-intervention group. There was no long-term difference in hip displacement. | |
| Pountney, 2002 |  | Chailey levels of ability (lying, sitting, standing) |  | MP >33%=subluxed, >80% =dislocated | Children using a 24-hour Chailey Adjustable Postural Support (CAPS) systems in 3 positions-lying, sitting and standing -before hip subluxation maintained significantly more hip integrity than other CAPS in sitting only or CAPS in lying/sitting or sitting/standing supports | |
| Pountney, 2009 |  | Chailey levels of ability (lying, sitting, standing) |  | MP 33-80% = subluxed, >80% =dislocated | Early provision of postural management equipment helps reduce the number of hip problems at 5 years of age. AM: confounders that increase uncertainty with this conclusion: historical control group, use of categorical rather than non-continuous data (actual differences might be small), other interventions (stretching, physiotherapy etc) not recorded. | |
| Rodriguez, 2010 | ROM (hip flexion, abduction, maximum separation of both thighs); adductor and hamstring spasticity (Tardieu Scale and Modified Ashworth Scale) |  |  | Reimer’s MP 10-40% | Botox was effective in reducing muscle tone and slowed progression of hip subluxation of during 3 months the drug is active. | |
| Rolauffs, 2007a | ROM (hip abduction) | Rancho Los Amigos classification (psychomotor abilitie) |  | Reimer’s MP | In cerebral palsy children with bilateral hip dislocation, conservative treatment of the less affected hip is suitable to achieve clinical and radiological results that are equal to the surgical treatment of the more affected hip. Combined conservative and surgical treatment of bilateral hip dislocation leads to an adequate motor function improvement that is comparable to established bilateral treatments | |
| Rolauffs, 2007b | ROM (hip abduction) | Rancho Los Amigos classification (psychomotor abilitie) |  | Reimer’s MP | For the most severe preoperative abduction restriction, improvement was most pronounced after the first year; the other groups showed less improvement. Higher MP preoperatively was associated with a greater postoperative improvement. | |
| Roposch, 2005 | Pain | Bespoke questionnaire to caregivers |  | MP <33% was normal, 33-99%= subluxed, 100%= dislocated.  Acetabular Index  Sharp angle | Mean AI improved from 32° preoperatively to 12° at follow-up. Mean MP improved from 77% to 13%. A redislocation or unstable hip occurred in two patients. According to caregivers, surgery improved personal care, positioning and comfort | |
| Rutz, 2012 | Gait Profile Score, Movement Analysis Profile (measured with 3DMA) |  |  | Hip dysplasia as MP >30%. Centre edge angle, Sharp angle, pelvic obliquity and Melbourne CP Hip Classification | Unilateral surgery including a proximal femoral osteotomy improved gait and walking kinematics in individuals with spastic hemiplegic CP, but hip dysplasia persisted | |
| Schejbalova, 2009 | Pain, ROM |  |  | NR | Schanz valgus osteotomy improves hip range of motion, relieves pain, and facilitates care of the patient. It is less invasive compared to proximal femoral excision and should preferably be used in older children in whom reconstructive surgery is not indicated | |
| Silverio, 2016 | Pain, ROM (categorical) | Caregiver satisfaction |  | Average percentage of prosthesis migration. Location of prosthesis at time of final follow-up, classified as infra-acetabular level (lower 1/3, middle 1/3, or upper 1/3 of the acetabulum}, and supraacetabular | Proximal femur prosthetic interposition arthroplasty (PFIA) is a salvage option for the painful, spastic dislocated hip. There were variable results with in pain and ROM outcomes post PFIA. The majority of caregivers were satisfied with the outcome and would recommend PFIA. | |
| Terjesen, 2019 | Pain |  |  | MP <33% =normal, 33-89% = subluxed, >90%= dislocated | Better primary correction was obtained with combined femoral and pelvic osteotomies than isolated femoral osteotomy, indicating that combined osteotomies are preferred method for hips with the most severe degrees of displacement. Significant risk factors for a poor final outcome were GMFCS level 5 and high MP 1-year postoperatively | |

3DMA, 3 dimensional motion analysis; ADLS, Activities of Daily Living; AI, Acetabular Index; CE, Centre Edge; CP, Cerebral Palsy; GMFCS, Gross Motor Classification System; ICF, International Classification of Functioning, Disability and Health; MP, Migration Percentage; NR, Not Reported; ROM, Range of Motion
