# Supplementary material for: Longitudinal relationship between hip displacement and hip function in children and adolescents with cerebral palsy: A scoping review
Source: Dev Med Child Neurol. 2024 Nov 21;67(4):450–62. doi: 10.1111/dmcn.16175 (PMC11875528; doi:10.1111/dmcn.16175)
Supplement: Supplementary file 3 — Table S3: Study characteristics with detail of study interventions. [file DMCN-67-450-s004.docx]

**Supplemental Table 3: Study characteristics with detail of study interventions**

| **Author, Year** | **Country** | **Setting** | **Study Design** | **Total Number of Participants** | **Number of hips** | **Number of Relevant Participants** | **GMFCS level** | **Mean age/Age range** | **Intervention** | **Follow up period** |
| --- | --- | --- | --- | --- | --- | --- | --- | --- | --- | --- |
| Abdo, 2016 | Brazil | Orthopaedic | Retrospective | 17 | 17 | 17 | IV and V | Mean 6.8 years. Range: 2.34-13.25 years | Surgery: Unilateral reconstruction surgery for hip dislocation (addjuctor release, femoral varus osteotomy and acetabuloplasty) | Mean 6.1 years. Range: 2.5-12.5 years |
| Al-Ghadir, 2009 | Canada | Orthopaedic | Retrospective | 39 | 52 | 39 | IV and V | Mean 8.1 yrs, 4.5-11.7 yrs | Surgery: Hip osteotomy | Median 53 mo (IQR 24.9 months) |
| Aly, 2019 | Egypt | Orthopaedic | Prospective case-series | 26 (11 female, 15 male) | 52 | 26 | IV and V | Range 4.0-14.0 yrs | Surgery: Femoral varus derotation osteotomy | Mean 36.8 mo, Range 2-4 yrs |
| Atar, 1995 | USA | Orthopaedic | Retrospective | 36 (14 female, 12 male) | 64 | 36 | NR | Range 2-15 yrs | Surgery: Femoral varus derotation osteotomy | Mean 3.8 yrs (range 3-6 yrs) |
| Bertoncelli, 2021 | France | Surveillance | Longitudinal, retrospective multicentre, double-blinded | 102 (42 female, 60 male) | 102 (42 female). 60 were inpatients and 42 were outpatients | 102 | III-V | Range 15.3-17.7 yrs | Botulinum toxin injection in adductor muscles (n=17), additional hip flexor injection (n=2). Hip surgery; (n=28, femoral osteotomy (n=22), additional pelvic osteotomy (n=11). Bilateral procedure (n=5). Multiple tenotomies (n=25) | Once a yr for 12 yrs |
| Boyd, 2001 | Australia | CP clinics | RCT | 39 (15 female, 24 male) |  | 39 | II-V | Range 1.6-4.8 yrs | Botulinum toxin injection to adductors and medial hamstrings, plus variable hip abduction orthosis (SWASH). Both groups received current clinical practice | One yr |
| Cho, 2018 | South Korea | Rehabilitation | Retrospective | 57 (26 female, 31 male) |  | 57 | I-V | Range 2.0-6.0 yrs | Botulinum toxin injection | Range 5-10 yrs |
| Cobanoglu, 2017 | Turkey | Orthopaedic | Retrospective | 30 (14 female, 16 male) | 45 | 30 | I-V | Range 5.0-18.0 yrs | Surgery: Pelvic and femoral osteotomy (7 dislocated hips and 8 subluxed hips). Femoral osteotomy (4 dislocated hips, 19 subluxed hips and 7 risky hips). | Range 2-11 yrs.  Mean 57 mo. |
| Cobeljic, 2009 | Serbia | Orthopaedic | Retrospective | 42 (21 female, 21 male) | 57 | 42 | I-III | Range 2.0-10.0 yrs | Surgery Group A: Iliopsoas tenotomies of subluxed hip, bilateral adductor tenotomies (n=20).  Group B: Same as Group A + Rectus femoris tenotomy and iliac crest resection (n=22) | Range 3-18 yrs |
| DiFazio, 2016 | USA | CP clinics | Prospective | 38 (15 female, 23 male) | NR | 38 | IV and V | Mean 10.1 (SD 3.90) yrs | Reconstructive hip surgery to correct severe spastic hip dysplasia | 6 weeks, 3, 6, 12 and 24 mo post-op |
| Hagglund, 2007 | Sweden | Surveillance | Longitudinal Registry study | 212 (gender NR) | NR | 212 | I-V | Range 2.0-9.0 yrs | None | 9-16 years. 2 follow-up per year age 2-6, 1x per year thereafter |
| Khot, 2008 | Australia | Combined surgical and medical | Prospective cohort pilot study | 16 (7 female, 9 male) | 32 | 16 | III and IV | Range 2.0-6.0 yrs | Combined medical and surgical interventions. Surgical lengthening of adductor longus and gracilis combined with phenolisation of the anterior branch of the obturator nerve. Botulinum toxin injection of hamstrings and calves | 2 yrs after surgery (ROM every 3 mo, X-rays every 12 mo) |
| Krebs, 2008 | Austria | Orthopaedic | Retrospective | 51 (gender NR) | 63 | 51 | I-V | Mean 8 yrs, 6 mo; Range 1 yr 8 mo-19 yrs 7 mo | Surgery: Salter innominate osteotomy (25 hips), Pemberton osteotomy (39 hips), intertrochanteric varus derotation and shortening. Osteotomy (58 hips). Additional soft tissue surgery (hip adductors and flexors) in 52 hips. | Mean 4 yrs, 10 mo, (range 11 mo-11 years, 10 mo) |
| Larsen, 2021 | Norway | Surveillance | Registry | 67 (28, female, 39 male |  | 67 | IV and V | Mean age 14yrs 7mo (SD 1y 5mo), Range 12–17 yrs | None | 3 yrs 8 mo-5 yrs 11 mo |
| Lee, 2021 | South Korea | Rehabilitation | Prospective | 20 (6 female, 14 male) |  | 20 | IV and V | Mean 5 yrs 1 mo (SD 1 yr, 10 mo). Range 2.0-10.0 yrs | Botulinum toxin  injection into adductor muscles at baseline and 6-month follow-up | 1,2,3,7 and 12 months |
| Martinsson, 2011 | Sweden | Rehabilitation | Prospective case-series | 97 (48 female, 49 male) | 97 (hip with highest MP included) | 97 | III-V | Range 2.0-6.0 yrs | Straddled standing (maximum tolerated hip abduction and extension, and knee extension (n=14) or in standing without abduction (n=63) for 1.5h x 1-3/day over 1-year. Surgery (n=14/97); n=3 from straddled intervention vs n=20 in non-straddled standing intervention | 1 yr |
| Martinsson, 2021 | Sweden | Surveillance (longitudinal retrospective case-control study) | Rehabilitation | 269 (118 female) | 269 (2 control groups n=240, CG1=after APT surgery (n=80), CG2=no surgery.  2 study groups (SG1: >10° abd standing after APT surgery (n=13), SG2: >10° abd standing, no surgery (n=16). | 269 | IV and V | Median age 3.7 yrs; Range 0.6-16 yrs | Use of stander or standing shell for 10 hours/week every week for 8 months-7 years.  Study group 15-30° abduction and control group 0-10° abduction | Median 1.5 (0.8-7.5) yrs from baseline, Median 3.5 (0.5 - 8.7) yrs from intervention |
| Moreau, 1995 | Canada | Orthopaedic | Prospective | 22 (10 female, 12 male) | 44 | 22 | IV and V | Mean 4 yrs, Range 2.0-6.0 yrs | Adductor longus and gracilis tenotomies and psoas tendon release | Yearly for radiographs and videos, at 2 and 5 years for questionnaires |
| Park, 2014 | South Korea | Surveillance | Retrospective | 48 | 90 | 48 | III-V | Mean 4.5 yrs | SG: Obturator Nerve block  CG: No block or Botulinum Toxin injection | Mean (SD) duration of follow-up : CG 20.10 mo (5.60), SG: 18.46 mo (6.50) |
| Pountney, 2002 | UK | Rehabilitation | Retrospective | 59 | 118 | 59 | NR | Range 5 mo- 9.8 yrs | 24-hr postural management using Chailey Adjustable Postural Support (CAPS)  SG 1: 3 postures: lying, sitting and standing.  SG2: 2 postures: lying/sitting or sitting/standing  SG3: CAPS seat only. | 1.2 to 16.9 years |
| Pountney, 2009 | UK | Rehabilitation | Prospective cohort study | 39 (16 female, 23 male) | 78 | 39 | III-V | NR. All <1.5yr at enrolment. Mean age at 30 mo follow-up=2.6yrs. Mean age at 5yr follow-up=5.1yrs. | 24-Hr Chailey postural management- 3 postures: lying, sitting and standing | 30 and 60 mo |
| Rodriguez, 2010 | Spain | Orthopaedic | Prospective | 10 (4 female) | 17 | 10 | III-V | Mean 5.5 years (SD 2.27 years) | BoNT-A injection | 1, 3 and 6 months post BoNT-A injection |
| Rolauffs, 2007a | Germany | Orthopaedic | Prospective | 91 (46 female) | 141 | 41 | NR | Mean 4 years, 11 months, Range: 1.1- 15.8 years | Physiotherapy, surgery | 1, 2, 3 and 4 yrs |
| Rolauffs, 2007b | Germany | Orthopaedic | Prospective | 91 (46 female) | 141 | 91 | NR | Mean age 4.9 years. Range: 1.1-15.8 years | Surgery | 1, 2, 3 and 4 yrs |
| Roposch, 2005 | Canada | Orthopaedic | Retrospective | 32 (14 female) | 41 | 32 | IV and V | 5.2-16.8 yrs | Surgery: Modified periacetabular osteotomy | Mean 5.3 yrs (range 2-11.7 yrs) |
| Rutz, 2012 | Australia | Orthopaedic | Retrospective | 11 (5 female, 6 male) | 11 | 11 | I and II | Range: 7-16 yrs | Surgery: Unilateral or multilevel surgery including external rotation osteotomy of the proximal femur | 2 yrs 3 mos- 10 yrs 8 mos |
| Schejbalova, 2009 | Czech Republic | Orthopaedic | Retrospective | 35 (gender NR) | 55 | 35 | IV and V | Range 9-18 yrs | Surgery: Palliative Schanz proximal femoral valgus osteotomy | Radiography at 3, 6 and 12 months, clinical follow-up yearly. Mean follow-up 98 +/- 4.5 mo |
| Silverio, 2016 | USA | Orthopaedic | Retrospective | 12 (3 female, 9 male) | 16 | 12 | V | Range 9.0-18.0 yrs | Surgery: Proximal femur prosthetic interposition arthroplasty (PFIA) | 24-60 mo |
| Terjesen, 2019 | Norway | Surveillance | Prospective | 31 (11 female, 20 male) | 39 | 31 | IV and V | Range 2.2-9.9 yrs | Femoral varus  osteotomy (13 patients, 20 hips) and combined Dega-type pelvic osteotomy and femoral osteotomy (18 patients, 19 hips). Bilateral tenotomies of adductor longus, gracilis and iliopsoas (23 patients) | 3.8-11 yrs |

APT, Adductor-Psoas Tenotomy; CAPS, Chailey Adjustable Postural Support (CAPS); CG, Control Group; IQR, Interquartile Range; mo, months; NR, Not Reported; PFIA, Proximal femur prosthetic interposition arthroplasty; RCT, Randomised Controlled trial; SD, standard deviation; SG; Study Group; yrs, years;
