# Supplementary material for: Longitudinal relationship between hip displacement and hip function in children and adolescents with cerebral palsy: A scoping review
Source: Dev Med Child Neurol. 2024 Nov 21;67(4):450–62. doi: 10.1111/dmcn.16175 (PMC11875528; doi:10.1111/dmcn.16175)
Supplement: Supplementary file 4 — Appendix S1: MEDLINE search strategy. [file DMCN-67-450-s002.docx]

**Appendix S1: Medline Search Strategy**

((((cerebral palsy[MeSH Terms]) OR (cerebral palsy[Title/Abstract] OR spastic diplegia[Title/Abstract]))

AND

((hip dislocation[MeSH Terms]) OR (hip[Title/Abstract] AND (dislocat*[Title/Abstract] OR subluxat*[Title/Abstract] OR luxat*[Title/Abstract] OR disarticulat*[Title/Abstract] OR surveillance[Title/Abstract]))))

AND

((longitudinal studies[MeSH Terms]) OR ((study[Text Word] OR studies[Text Word]) AND (longitudinal[Text Word] OR prospective[Text Word] OR retrospective[Text Word] OR cohort[Text Word]))))

AND (((adolescent[MeSH Terms]) OR (infant[MeSH Terms])) OR (child[MeSH Terms]))
