# Supplementary figures and images for: Longitudinal relationship between hip displacement and hip function in children and adolescents with cerebral palsy: A scoping review
Source: Dev Med Child Neurol. 2024 Nov 21;67(4):450–62. doi: 10.1111/dmcn.16175 (PMC11875528; doi:10.1111/dmcn.16175)

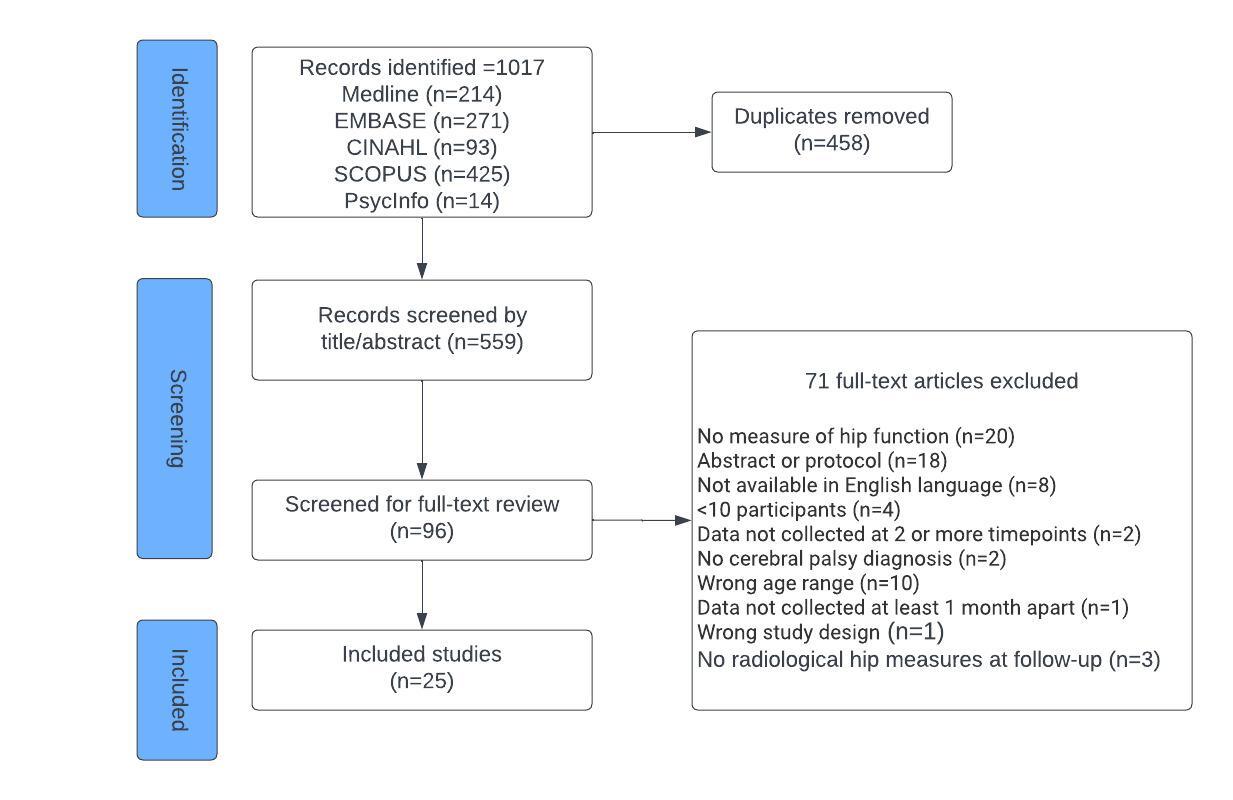

Supplement: Supplementary file 5 — Figure S1: Preferred Reporting Items for Systematic reviews and Meta‐Analyses extension for Scoping Reviews (PRISMA‐ScR) flow diagram. [file DMCN-67-450-s003.jpeg]
